# Influence of valsartan-eluting stent on neointima formation

**DOI:** 10.4103/0975-3583.59980

**Published:** 2010

**Authors:** Li Guihua, Lei Wang, Jia Sanqing, Wenlin Ren, Zhao Lin, Yao Daokuo, Ding Rongjing

**Affiliations:** * *Cardiovascular center of Chuiyangliu Hospital, Beijing, PRC, 100022 China*; *Cardiovascular center of Capital University of Medicine Science Affiliated Friendship Hospital, Beijing, 100050, China*; # Department of Cardiology, Cardiovascular Disease Insitution, Peking University People’s Hospital, Beijing 100044, PRC, China

**Keywords:** *eluting stent*, *valsartan*, *restenosis*, *collagen*, *AT2 receptor*

## Abstract

**Objective :—:**

This study is to explore the effect of valsartan-eluting stents on neointima formation after stenting and to elucidate possible mechanisms how locally used valsartan prevents in-stent restenosis (ISR). METHOD: valsartan- and carriereluting stents were manufactured by using multi-layer-coated technology. Bare stents, carrier-eluting stents and valsartan– eluting stents were implanted into the abdominal aortas of the rabbits respectively. Quantitative angiography (QA) before, immediately after and 3 months after stent implantation were compared between the groups of bare (n=8), carrier-eluting (n=8) and valsartan-eluting stent (n=10), which allows the comparison of vascular diameters of aortas as well as indices of vascular neointimal formation, i.e. luminal area (LA), neointimal area (NIA), inner elastic membrane luminal area (IELA) and the maximal inner-membrane thickness (MIT) in 15 rabbits. α-Actin protein expression were detected by Envision two-step immunohistochemistry. Mean positive indices (MPI) of the above protein were analyzed semi-quantatively by IMS(Information Management System) cell image analysis system. MPI=positive area×OD (optical density). Collagen deposition in neointima was observed through MASSON stain among the three groups.

**Result:—:**

the mean aortic diameters were similar in the three groups:bare stents group(n=8), carrier-eluting stents group(n=8) and valsartan eluting stents group(n=10) measured by QA at different time. A larger luminal area and a less neointimal hyperplasia in valsartan eluting-stents group was found compared with the other two groups. The mean luminal areas were 4345548±125822um^2^; 4302061±167952 um^2^; 5016269±207934um^2^ respectively. The mean neointimal areas were 1119635±163503um^2^; 1135636±136555um^2^; 441577±74099um^2^ and the mean maximal inner-membrane thickness were 210±30um;192±21um; 116±12um respectively. α-Actin protein expression was significantly lower in neointima of valsartan eluting-stents group than the other two groups. Through MASSON stain we found that Collagen was much richer in neointima of bare stents group and carrier-eluting stents group than valsartan eluting-stents group.

**Conclusion:—:**

Valsartan eluting-stents inhibited neointimal hyperplasia after stenting by decreasing collagen deposition and smooth muscle cell proliferation. Therefore it would be potentially effective in preventing in-stent restenosis.

**Abbreviations:—:**

Quantitative angiography (QA), luminal area (LA), neointimal area (NIA), inner elastic membrane luminal area (IELA), the maximal inner-membrane thickness (MIT), Mean positive indices (MPI), optical density (OD), Drugeluting stents (DES), in-stent restenosis(ISR), percutaneous transluminal coronary angioplasty (PTCA), angiotensin α type 2 receptor (AT2).

## BACKGROUND

With the extensive use of stents in-stent restenosis is becoming a major drawback. A series of intravascular studies confirm that elastic recoil and negative remodeling which caused restenosis after PTCA mainly is counteracted by stents, so in-stent restenosis is mainly caused by intimal hyperplasia. Recently, impressive results have emerged in the field of ISR prevention. Drug-eluting stents (DES) coated with the strong antiproliferative agents rapamycin or paclitaxel have been demonstrated to be potent antirestenotic strategies[1,2]. Notwith- standing this tremendous progression in antirestenotic therapies, with the use of DES in the “real world,” target vessel revascularization remains necessary in approximately 4%[3]. Moreover, some concerns have emerged about the occurrence of late thrombosis and hypersensitivity reactions after DES implantation[4] Consequently, refinement of antirestenotic therapies remains necessary. Recently, there is an increasing interest in physiological antirestenotic therapies by means of restoring the normal biologic function of the vessel wall. A lot of fundamental studies have proved that Ang II accelerates restenosis through acting with the growth factors. AT1 receptor antagonists can reduce restenosis through blocking the combination of Ang II and AT1 receptor, reducing the concentration of growth factors related with restenosis and improving the concentration of growth factors inhibiting restenosis. Val-PREST and VALVACE trial have confirmed that oral administration of valsartan reduce restenosis rate after stenting[8,9] but the mechanism how locally used valsartan prevents restenosis remains unclear.

## OBJECTIVE

To explore the effect of valsartan eluting stent on intimal hyperplasia after stenting and elucidate the possibility and mechanism of valsartan eluting stent to prevent in-stent restenosis.

## METHODS

### Experimental animals and groups

15 adult New Zealand white rabbits (ignorance of gender, weighting 2.75-3.25kg, provided by Beijing Friendship Hospital animal laboratory) were acclimatized to the animal quarters for at least one week. They were divided into bare-mental stent, carrier-eluting stent and valsartan-eluting stent group according to the stent types. There were 5 in each group.

### 2. Equipment and implantation of stent:

30 316L stainless steel stents with length 10mm and diameter 3.0mm were chosen, among which 20 stents were manufactured into eluted, i.e. 10 valsartan-eluting stents in which Valsartan was embedded in base coating in a dosage of hundreds ug/cm^2^; and another 10 carrier-eluting stents, by using the exactly same multi-layers coated technique, while 10 were left to be bare mental stents. According to the multi-layers coated technique, the eluted stents were manufactured with base coating, which is contented with organic-inorganic compound, and top coating, which is made with high compatible polymer full of phospholipid groups. The only difference of the two different eluted stents is that Valsartan was embedded in base coating during the manufacture in the valsartan-eluting stent, while the carrier-eluting stent was not. The animals were anesthetized celiacly with Ketamine, the right common femoral artery was surgically exposed and the adventitia circumferentially cleared. A 4-French arterial sheath was introduced through the incision up to the aorta. Meanwhile, heparin in a dosage of 100IU/kg was injected through the sheath. An angioplasty balloon (3.0^*^20mm) with stent was introduced by guiding wire (0.014”×190cm) in the superficial femoral artery and advanced into the common femoral artery to a standardized location just distal to the bifurcation of the renal artery. The balloon was inflated to 12–14 atm in 30–60 seconds before it was withdrawn and released the stent. The other stent was implanted in the same way and the distance between the stents was about 2 cm. The catheter was then removed, and the incision was closed after the implantation. Regular injection of 400000u Benzylpenicillin Sodium was administered for 3 days in order to prevent infection. All the rabbits were raised for 3 months.

### 3. Evaluation methods and indices:

Angiographic analysis: Abdominal aorta converse angiography was performed before, immediate after and 3 months after stents implantation. Diameter, stenosis percent of diameter and area of stent section of vessel were analyzed by QA.

### Pathological analysis:

Three months later, the rabbits were scarified. The vessels with stents were taken out and put into formalin (4%) for fixation, then was embedded by stiff plastic. Slices were made by polycut slicemachine and were processed with HE, MASSON and picrosirius staining. Indices of the vascular neointimal formation, i.e. Inner and external elastic membrane luminal area, the maximal intimal thickness, neointimal area and stenosis area percent were analyzed by Leica Q550CW image analysis system in HE stain. Collagen deposition was analyzed by MASSON stain by light microscope. Collagen integral optical density was analyzed quantitatively by image analysis system.

### Immunohistochemistry:

α-Actin protein expression was detected by Envision two-step immunohistochemistry. Immunohistochemical staning was performed on formalin-fixed, paraffin embedded sections by Envision technique using the kit (Dako Corporation).

Mean positive indices(MPI) analyzed by IMS cell image analysis system was used to evaluateα-Actin semi-quantitatively. MPI=positive area×OD (optical density)

## RESULTS

### 1. Stent Implantation

Despite the death of a rabbit during the procedure of implantation, we succeeded in implanting 8 bare stens into 4 rabbits, 10 carrier-eluting stents into 5 rabbits(a rabbit died because of diarrhea later), 10 valsartan-eluting stents into 5 rabbits.

### 2. QA analysis

There was no difference between any two of the three groups in terms of the mean diameters of the celiac aortic at before, immediately after or 3 months after implantation by QA comparison. No in-stent restenosis (diameter stenosis >50%) was found in any group according to the repeated QCA at 3 months after implantation(data not shown here).

### 3. HE Stain Image Analysis of Slices

**Figure 1:**
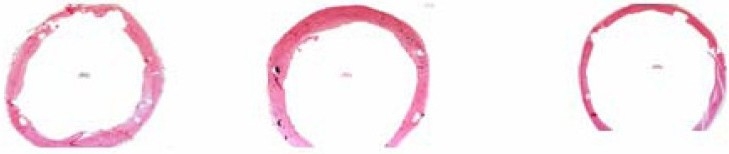
bare-mentel stent group; carrier-eluting stent group; valsartan-eluting stent group

It was indicated that a significantly larger luminal area (LA) and less neointimal hyperplasia was found in valsartan-eluting stent group compared with the other two groups. The mean LA of bare group, carrier eluting stent group and valsartan-eluting stent group were 4345548±125822um^2^, 4302061±167952 um^2^ and 5016269±207934um^2^ respectively. It was also found that the MIT in all three groups were significantly increased at 3 months after implantation. Furthermore, MIT and NIA in valsartan-eluting group were significantly smaller than those of the other two groups with a significantly larger LA as indicated in the table 1 and Figure 1.

**Table 1:** Results of HE stain

goups	ELA(um^2^)	IELA (um^2^)	LA (um^2^)	NIA (um^2^)	MIT(um)	PAS(%)
Bare	6406076±177475	5465184±215062	4345548±125822▲	1119635±163503▲	240±30▲	20 ±2.3▲
Carrier	6309791±176032	5437697±183248	4302061±167952▲	1135636±136555▲	192±21▲	21±2.3▲
Valsartan	6463743±195148	5457847±162519	5016269±207934	441577±74099	116±12	8±1.5

**Figure 2:**
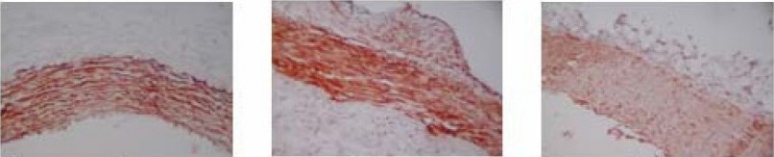
bare-mentel stent group; carrier-eluting stent group; valsartan-eluting stent group

α-Actin Immunohistochemistry:

α-Actin expressed in intimal and media in both bare-mental stent group and carrier-eluting stent group while it expressed a little fainter in valsartan-eluting stent group compared with the other two groups. The mean positive index by image analysis was 463993.2± 4298.0 vs 474118.3±15116.3 vs 232219.2±19713.8 in baremental stent group, carrier-eluting stent group and valsartan-eluting stent groups. There was siginificant differences compared valsartan eluting stent group with the other two groups (P<0.01) (Figure 2).

### 4. MASSON Stain Analysis:

**Figure 3:**
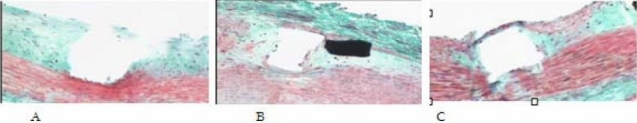
MASSON Stain A: bare-mental stent group; B: carrier-eluting stent group; C: valsartan-eluting stent group

A lot of collagen deposition was found in neointima in the bare-mental stent group and carrier-eluting stent group by MASSION stain but there was much less collagen found in neointima of valsartan-eluting stent group(Figure 3). Integral Optical Density of positive target analyzed by collagen image analysis system was 2292.5±179.5, 2346.2±119.7, 1320.0±103.8 respectively in bare mental stent group, EELA:external elastic laminal area, IELA: internal elastic laminal area, LA:luminal area;NIA :neointimal area, MIT: maximal inner-membrane thickness, PAS:percent of area stenosis compared with valsartan group p>0.05; ▲compared with valsartan group p< 0.01. carrier-eluting stent group and valsartan-eluting stent group. Compared with valsartan-eluting stent group Integral Optical Density in bare mental stent and carrier-eluting stent were much higher (p< 0.01). There was no obvious difference between the other two groups.

## DISCUSSION

ISR, mainly caused by intimal proliferation^5^ almost, is a major drawback of percutaneous transluminal coronary angioplasty with stent placement. There are two kinds of drug-eluting stents widely used clinically to reduce restenosis effectively: rapamycin-eluting stent and paclitaxel-eluting stent both rapamycin and paclitaxel- are strongly anti-proliferative agents, they can inhabit SMC proliferation and migration so inhabit neointimal formation and reduce restenosis. However, late thrombosis, inflammation, hypersensitivity reactions and endothelium healing delay are of major concern. In light of the recent interest in more physiological endothelium and arterial recovering antirestenotic strategy for the prevention of IRS, RAS intervention attracted more research interests. The RAS is not the sole factor for restenosis; However, it surely plays a part in the pathophysiology, RAS intervention would be an attractive way to prevent ISR in a more physiological manner. RAS intervention has endothelium-protective properties, even in the setting of vascular injury[6,7].

VAL-PREST[8] and VALVACE[9] trials have proved that systemically administered AT1 receptor blockade could reduce ISR. To study the effect of valsartan eluting-stents on vascular neointimal formation and assess the feasibility to prevent restenosis, we coated stents with Valsartan and carried out animals experiment. The results showed Valsartan eluting-stents inhibited neointimal hyperplasia and might play an important role in preventing restenosis.

## COLLAGEN AND RESTENOSIS

More recently, extracellular matrix accumulation has been recognized as a very important component of in-stent restenosis in the chronic phase after stent implantation. A substantial portion of the neointima consists of extracellular matrix rather than cells[10]. From 2 to 6 months, stented porcine coronary arteries show a reduction in neointimal hyaluronan associated with reduced neointimal type III and increased type I collagen[11]. Observations to a more chronic phase of neointimal evolution have been extended to more than 18 months and found that the neointima in stents in place up to18 months remains hypercellular (confluent SMCs) and rich in type III collagen, versican, and hyaluronan with relatively little type I collagen and decorin. In contrast, stents ≥18 months old demonstrated weaker staining for versican, hyaluronan, and type III collagen and stronger staining for type I collagen and decorin. Neointimal cell density, area occupied by SMCs, and in-stent stenosis were smaller in stents≥18 months old group compared with stents<18 months old group. These data demonstrate that intimal lesions at 18 months after stenting resemble wounds that are not fully healed and suggest that neointimal retraction occurs subsequently.[12]

### 

#### RAS and RESTENOSIS

It is known that there are 4 subtype angiotensin II receptors:AT1, AT2, AT3 and AT4. RAS exerts its physiological effects through Angiotensin II stimulating angiotensin II receptors, especially AT1 and AT2. Activation of the AT1 receptor has deleterious effects on the cardiovascular system, namely cell migration and proliferation, extracellular matrix deposition, inflammation, promotion of thrombosis, and production of reactive oxygen species. Activation of the AT2 receptor could counteract effects of the AT1 receptor Activating. Activation of the AT2 receptor is associated with NO production, inhibition of proliferation, and induction of apoptosis. Selective AT1 receptor blockers inhibit stimulation of AT1 receptor by Angiotensin | |, the AT2 receptor can still be activated. So adverse effects of Angiotensin |, such as cell migration and proliferation, extracellular matrix deposition, inflammation, promotion of thrombosis, and production of reactive oxygen species, are blocked, and the advantageous effects of AT2 activation still remain. AT1 receptor and AT2 receptor interacts with each other. Wu et al[13] elucidated the function of the AT2 receptor while AT1 receptor antagonism after vascular injury. Neointimal formation was attenuated in wild mice by AT1 receptor blockade; this effect was less prominent in AT2 knockout mice. Moreover anti-inflammatory effects of AT1 receptor blockade were decreased in AT2 knockout mice. The conclusion was that stimulation of the AT2 receptor during AT1 receptor antagonism is important in the decrease of neointimal formation after vascular injury. Furthermore, Suzuki et al[14] demonstrated neointimal formation as well as DNA synthesis in vascular smooth cells after vascular injury was exaggerated in AT2 knockout mice, but they were both suppressed in AT1 knockout mice compared with those in wild-type mice. In contrast the number of apoptosis cells in the injured artery in VSMC was significantly increased in AT1 knockout mice but decrease in AT2 knockout mice. The result suggests AT2 exerts antiproliferative effects and propoptotic changes in VSMC by counteracting AT1 in the process of neointimal formation after vascular injury. Our study shows that valsartan-eluting stents, local application of valsartan, could inhibit neointimal formation, it may be an attractive methods to prevent ISR.

### Funding sources

*The project was funded by Beijing municipal natural science fund (NO. 7042021)*.

### Conflict of interest

None. The authors declare that they had no financial or personal relations to other parties whose interests could have affected the content of this article in any way, either positively or negatively.
